# Artificial intelligence-driven reverse vaccinology for *Neisseria gonorrhoeae* vaccine: Prioritizing epitope-based candidates

**DOI:** 10.3389/fmolb.2024.1442158

**Published:** 2024-08-13

**Authors:** Ravi Kant, Mohd. Shoaib Khan, Madhu Chopra, Daman Saluja

**Affiliations:** ^1^ Medical Biotechnology Laboratory, Dr. B. R. Ambedkar Center for Biomedical Research, University of Delhi, Delhi, India; ^2^ Delhi School of Public Health, Institute of Eminence (IoE), University of Delhi, Delhi, India; ^3^ Laboratory of Molecular Modeling and Anticancer Drug Development, Dr. B. R. Ambedkar Center for Biomedical Research, University of Delhi, Delhi, India

**Keywords:** hypothetical proteins, vaccine, epitopes, functional annotation, sub-cellular localization, immuno-informatics, antigenicity, *Neisseria gonorhoeae*

## Abstract

*Neisseria gonorrhoeae* is the causative agent of the sexually transmitted disease gonorrhea. The increasing prevalence of this disease worldwide, the rise of antibiotic-resistant strains, and the difficulties in treatment necessitate the development of a vaccine, highlighting the significance of preventative measures to control and eradicate the infection. Currently, there is no widely available vaccine, partly due to the bacterium’s ability to evade natural immunity and the limited research investment in gonorrhea compared to other diseases. To identify distinct vaccine candidates, we chose to focus on the uncharacterized, hypothetical proteins (HPs) as our initial approach. Using the *in silico* method, we first carried out a comprehensive assessment of hypothetical proteins of *Neisseria gonorrhoeae,* encompassing assessments of physicochemical properties, cellular localization, secretary pathways, transmembrane regions, antigenicity, toxicity, and prediction of B-cell and T-cell epitopes, among other analyses. Detailed analysis of all HPs resulted in the functional annotation of twenty proteins with a great degree of confidence. Further, using the immuno-informatics approach, the prediction pipeline identified one CD8^+^ restricted T-cell epitope, seven linear B-cell epitopes, and seven conformational B-cell epitopes as putative epitope-based peptide vaccine candidates which certainly require further validation in laboratory settings. The study accentuates the promise of functional annotation and immuno-informatics in the systematic design of epitope-based peptide vaccines targeting *Neisseria gonorrhoeae*.

## Graphical Abstract

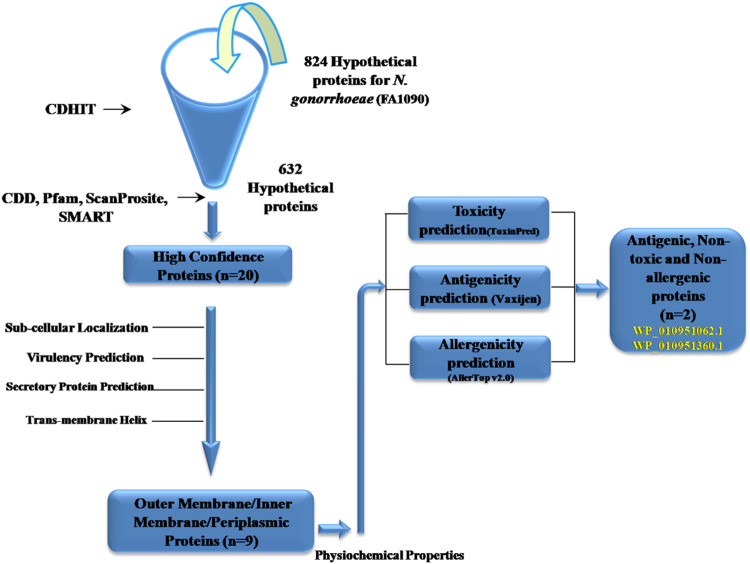

## Introduction

Gonorrhea, caused by the bacterium *Neisseria gonorrhoeae*, stands as a persistent global health challenge, underscored by its escalating prevalence and the concerning emergence of antibiotic-resistant strains (Jefferson et al., 2021). The lack of a viable vaccine against this sexually transmitted infection further augments the complexities in disease management. As traditional treatment options confront diminishing efficacy, it necessitates a paradigm shift towards preventive measures through vaccination (McIntosh, 2020). Within the context of *N. gonorrhoeae*, our study addressed the urgent need for a vaccine by presenting an *in silico* approach that integrates two crucial components: functional annotation of hypothetical proteins and immuno-informatics predictions of potential vaccine candidates.

Hypothetical Protein (HP) is the term used when a protein is assumed to be encoded by a well-defined open reading frame (ORF), but no experimental protein product has been identified or characterized (Galperin and Koonin, 2004). The majority of genomes contain approximately fifty percent of the HPs with proteomic and genomic significance (Nimrod et al., 2008). These HPs are believed to play crucial roles in the pathogen’s survival and disease progression. Through accurate annotation of these HPs new pathways, structures, and function cascades can be identified, and novel HPs can serve as a marker or target for vaccine or drug development applications (Desler et al., 2009). More than 800 proteins of *N. gonorrhoeae* FA 1090 strain are unknown in terms of their functions and biochemical characteristics.

The hypothetical proteins (HPs) of numerous bacteria, such as *Rhodobacter capsulatus* (Mondol et al., 2022), *Streptomyces coelicolor* (Ferdous et al., 2020), *Chlamydia trachomatis* (Li et al., 2011), *Haemophilus influenza* (Shahbaaz et al., 2013), etc. have been thoroughly studied in previous bioinformatics research utilising structure and sequence-based approaches. To the best of our knowledge, there have not been any comparable studies on *N. gonorrhoeae*. As a result, this study is a groundbreaking attempt to thoroughly analyse the roles and structures of preserved HPs unique to *N. gonorrhoeae*. By performing this analysis, we hope to better understand the potential roles and significance of these HPs in the pathogenesis and survival of *N. gonorrhoeae*, which will help us to identify potential vaccine candidates and develop new therapeutic approaches.

The landscape of vaccine development has undergone transformative changes with the integration of cutting-edge technologies, prominently featuring Artificial Intelligence and Machine Learning (ML), within the framework of reverse vaccinology (Kaushik et al., 2023). Harnessing the power of bioinformatics tools, advanced algorithms, and comprehensive genome analysis, *in silico* approaches offer a promising avenue for systematically identifying potential vaccine candidates. By intricately scrutinizing the genomes of pathogens, these methodologies unveil critical targets for immunogenicity, thereby enhancing the precision and efficiency of the vaccine development process (Motamedi et al., 2023).

This approach builds upon foundational research that highlights the pivotal role of antigenic epitopes in eliciting robust immune responses against pathogens (Habib et al., 2024). Our methodology involves a detailed analysis of hypothetical proteins in *N. gonorrhoeae* genome, employing sophisticated AI-powered techniques for annotation, characterization, and identifying conserved domains and structural features essential for the pathogen’s survival (Mazumder et al., 2022). Furthermore, we investigated physicochemical properties, sub-cellular localization, allergenicity, antigenicity and virulence factors, to establish correlations between functional significance and immunogenic potential, leveraging AI-enabled approaches in bioinformatics (Chen et al., 2021).

The integration of epitope prediction methods for B-cell and T-cell epitopes represents an additional layer of analysis. Advanced AI-driven algorithms which are instrumental in epitope prediction, provide an intricate understanding of the immunogenicity of identified proteins, optimizing the selection of promising vaccine candidates (Ponomarenko et al., 2008; Sanchez-Trincado et al., 2017). By utilizing these multifaceted analyses, our *in silico* strategy, aspires to strategically prioritize prospective vaccine candidates against *N. gonorrhoeae*, laying the foundational groundwork for subsequent rigorous experimental validation.

## Results

### Evaluation of hypothetical protein from *Neisseria gonorrhoeae* genome

Using data retrieved from the NCBI database, we identified 890 hypothetical protein sequences within the *N. gonorrhoeae* genome. To minimize redundancy and obtain a high-quality dataset for further analysis, we implemented a filtering procedure detailed in the methods section. This initial step yielded a set of distinct protein sequences (*n* = 824). Subsequently, CD-HIT, a bioinformatics tool adept at eliminating highly similar sequences, was employed (with default settings such as default sequence identity cut-off = 0.9) to further refine the dataset. This step resulted in 632 clusters and we retained one sample sequence from each cluster, giving a final set of unique protein sequences (*n* = 632). These refined sequences were then subjected to a sequential filter of bioinformatics tools (Supplementary Table S1) to predict their functional characteristics relevant to *N. gonorrhoeae* pathogenesis.

### Great degree of confidence (GDC) protein subset

To ensure high confidence in the predicted protein functions, a stringent filtering step was implemented. All 632 protein sequences were analyzed using five bioinformatics tools: CDD-BLAST, SMART, PFAM, ScanProsite, and InterProScan. Only proteins with consistent functional predictions across all five tools were retained for further investigation. This strategic approach yielded a final set of 20 proteins designated as “**Great Degree of Confidence**” **(GDC)** (Ezaj et al., 2021)**.** This substantial reduction from the initial set highlights the importance of this stringent filtering step in identifying a reliable group of twenty proteins for further studies aimed at elucidating their functional roles in the bacterium (Naorem et al., 2022; Supplementary Table S2).

### Functional annotation of great degree of confidence (GDC) proteins

**FIGURE 1:**
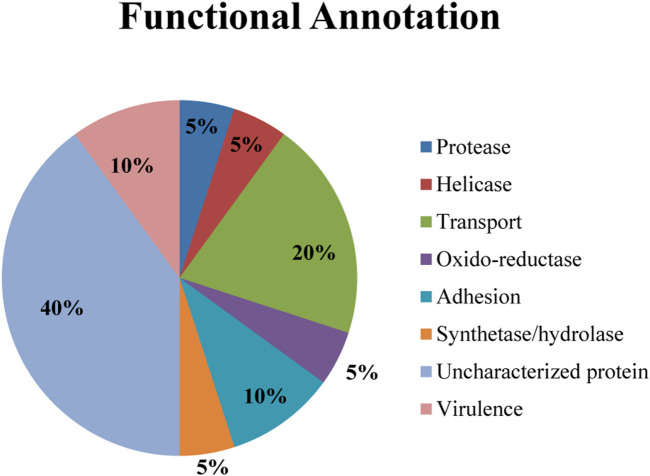
Functional annotation of twenty great degree confidence (GDC) proteins identified in *Neisseria gonorrhoeae*.

Analysis of the twenty identified great degree of confidence (GDC) proteins using bioinformatics tools (details in Methodology Section) revealed a diverse functional repertoire depicted in the pie chart (Figure 1). Notably, this chart represents the predicted functional distribution and categorization of these GDC proteins. The most prominent category within the GDC set comprised uncharacterized proteins (40%). These proteins lack currently assigned functions in existing databases. Further investigation using a combination of experimental and computational approaches is warranted to elucidate their roles in *N. gonorrhoeae* biology.

Beyond uncharacterized proteins, a range of functional categories were identified, potentially contributing to various aspects of physiology and pathogenesis by *N. gonorrhoeae*. Notably, the presence of proteins associated with virulence (10%) suggests their potential involvement in disease-causing processes. Transport proteins (20%) represent another significant category. These proteins facilitate the movement of molecules across the bacterial membrane, playing a crucial role in nutrient acquisition, waste removal, and potentially virulence factor secretion.

Other notable categories included Adhesion proteins (10%). These proteins mediate bacterial attachment to host cells, a critical step for colonization and pathogenesis. Oxido-reductases enzymes (5%) participate in electron transfer reactions, essential for bacterial metabolism and energy production. Proteases (5%) enzymes cleave protein bonds and may play a role in various cellular processes, including nutrient breakdown, protein turnover, and potentially virulence factor activation. Synthases/Hydrolases were also identified (5%) and may be involved in the synthesis and breakdown of various molecules, potentially contributing to cell wall maintenance, metabolite production, and other critical functions.

While further investigation is required to determine the specific functions of these GDC proteins, their diverse repertoire suggests potential roles in various aspects of *N. gonorrhoeae* biology and pathogenesis. Understanding these functions can provide valuable insights into the mechanisms employed by *N. gonorrhoeae* to establish infection and may contribute to the development of novel therapeutic strategies.

### Physicochemical characterization

The Expasy ProtParam server was used to calculate the physicochemical properties of these twenty GDC proteins. Table1 shows the theoretical iso-electric point (pI), molecular weight (MW), total amino acid number, Aliphatic index, extinction coefficient, grand average of hydropathy (GRAVY), total number of positively and negatively charged residues, and instability index. These GDC proteins had pI values that ranged anywhere from 5 to 11.29. The amount of light at a given wavelength that is absorbed by proteins is quantified by their extinction coefficient. The instability index provides a rough estimate of a protein’s stability *in vitro*. Protein with an instability index less than 40 is regarded as stable, while proteins with an instability index larger than 40 are deemed unstable. According to this standard, 11 proteins were found to be stable and 9 to be unstable.

**TABLE 1:** Physicochemical characterization of 20 shortlisted GDC proteins with Expasy ProtParam.

S.No.	Protein accession ID	UniProt ID	Proteins matched	No. of amino acids	MW	PI	Extinction coefficient	Instability index	Classification	Aliphatic index	GRAVY	Asp + Glu	Arg + Lys
1	WP_003690500.1	Q5FAK6	--	142	15444.06	7.68	12740	36.24	Stable	101.76	0.187	--	--
2	WP_010359741.1	A0A0H4ISI6	TonB-dependent receptor	71	7426.5	8.16	8480	55.01	Unstable	86.9	0.227	4	5
3	WP_003699500.1	A0A4D7WJT0	NAD(P)H:quinone oxido-reductase	126	14586.86	11.29	9190	59.64	Unstable	72.62	−0.811	8	27
4	WP_003687455.1	Q9JZX6	Uncharacterized protein	112	13206.92	9.75	33460	37.25	Stable	63.57	−0.761	12	17
5	WP_003690747.1	Q5F9W6	Uncharacterized protein	151	17410.69	8.35	23505	24.8	Stable	65.23	−0.587	17	19
6	WP_003687711.1	Q5F9R8	Uncharacterized protein	159	18002.62	6.31	21680	44.72	Unstable	101.76	−0.219	22	21
7	WP_003690844.1	Q5F9K2	Uncharacterized protein	188	20134.78	5.04	5500	47.82	Unstable	109.63	−0.127	27	22
8	WP_010357457.1	Q5F9F7	Outer-membrane lipoprotein LolB	193	21187.81	5.94	31065	44.56	Unstable	84.46	−0.38	20	18
9	WP_003689132.1	Q5F9B4	DNA helicase	453	49486.6	5.36	31650	41.45	Unstable	95.5	−0.228	63	53
10	WP_003706020.1	Q5F9A7	Uncharacterized protein	168	19127.25	9.61	13075	29.64	Stable	91.61	−0.299	17	26
11	WP_010951062.1	Q5F9A2	Caudovirus prohead protease family protein	385	40167.97	5	19940	28.72	Stable	73.95	−0.541	67	55
12	WP_010358353.1	Q5F7Z3	RelA_SpoT domain-containing protein	124	13766.66	5.32	13980	45.02	Unstable	93.63	−0.14	15	12
13	WP_225577510.1	Q5F7S3	Uncharacterized protein	755	83845.69	5.26	100060	43.67	Unstable	77.43	−0.44	102	86
14	WP_010951199.1	Q5F7K6	Zot domain-containing protein	361	40801.79	8.78	51715	37.23	Stable	72.11	−0.49	42	48
15	WP_003705520.1	Q5F7L5	Pertactin domain-containing protein	284	31433.26	8.88	46995	43.06	Unstable	66.58	−0.634	35	40
16	WP_010951229.1	Q5F6B6	Zot domain-containing protein	361	40741.69	8.78	51715	37.18	Stable	72.11	−0.5	42	48
17	WP_225577457.1	Q9JYB9	LPD3 domain-containing protein	161	18083.64	9.72	18575	21.55	Stable	73.29	−0.61	19	29
18	WP_232469644.1	Q5F6V0	YadA_head domain-containing protein	220	22656	5.67	11920	32.18	Stable	83.09	−0.162	23	20
19	WP_003701667.1	Q5F6A8	Bro-N domain-containing protein	280	30877.66	9.45	35535	36.95	Stable	94.14	−0.157	27	36
20	WP_010951360.1	A0A0H4J5Y2	Lipoprot_C domain-containing protein	353	38769.67	5.68	32320	34.48	Stable	73.99	−0.609	55	50

The percentage of a protein that is made up of aliphatic side chain amino acids is known as its aliphatic index. The aliphatic index values ranged from 63.57 to 109.63 for these twenty GDC proteins. The GRAVY score for a peptide or protein was determined by adding up the hydropathy scores of each amino acid and then dividing it by the total number of residues in the query sequence. The GRAVY scores of hydrophobic proteins are positive, while those of hydrophilic proteins are negative. Only two GDC proteins (WP_003690500.1 and WP_010359741.1) were identified to have positive GRAVY scores with values close to zero.

### Sub-cellular localization prediction

Using a variety of bioinformatics tools, the sub-cellular localization of the GDCHPs was predicted, along with their solubility, secretion or signaling ability, and potential membrane helices. Among the twenty GDCHPs, we predicted nine proteins (WP_010359741.1,WP_003690747.1, WP_010357457.1, WP_010951062.1, WP_010951199.1, WP_003705520.1, WP_010951229.1, WP_232469644.1, WP_010951360.1) that are in or near the outer membrane or periplasmic space of *N. gonorrhoea*e (Table 2). Out of these nine proteins, four (WP_010359741.1, WP_003690747.1, WP_010357457.1 and WP_010951360.1) proteins possess a consensus signal peptide, ‘a targeting signal’ guiding the protein to the appropriate location within the cell. Proteins with signal peptides are often directed to the endoplasmic reticulum (ER) or other cellular compartments involved in protein secretion (Supplementary Figure S1).

**TABLE 2:** Shortlisted proteins having non-cytoplasmic localization.

S. No.	Protein accession ID	Consensus location	Signal peptide	Secretory protein	Trans-membrane helix
1	WP_010359741.1	Inner Membrane	Yes	Yes	No
2	WP_003690747.1	Periplasmic	Yes	Yes	No
3	WP_010357457.1	Periplasmic	Yes	No	No
4	WP_010951062.1	Periplasmic	No	Yes	1TM
5	WP_010951199.1	Periplasmic	No	Yes	2TM
6	WP_003705520.1	Outer Membrane	No	Yes	Glob
7	WP_010951229.1	Periplasmic	No	Yes	2TM
8	WP_232469644.1	Outer Membrane	No	No	Glob
9	WP_010951360.1	Outer Membrane	Yes	Yes	1TM

Further analysis of these four proteins (WP_010359741.1,WP_003690747.1, WP_010357457.1 and WP_010951360.1) using SecretomeP 2.0 predicted these proteins to be secretary in nature. Hence, these proteins have the potential to be secreted or targeted to cellular membranes, indicating a potential role in extracellular or membrane-related processes. Protein segments called transmembrane regions move across the lipid bilayer of biological membranes. The membrane transport, signal transduction, and receptor activation are just a few of the actions that these regions are essential for in the body. Understanding a protein’s structure, function, and cellular localization depends on being able to identify its transmembrane sections. These four proteins (WP_010359741.1,WP_003690747.1, WP_010357457.1 and WP_010951360.1) were predicted as soluble proteins (SP) by DeepTMHMM web-server. It uses a deep learning approach to analyze protein sequences and find the existence and position of transmembrane sections. However, one protein (WP_010951360.1) out of these four was predicted to have a trans-membrane helix while the other three were predicted to have no trans-membrane helix as predicted by DeepTMHMM and were predicted as soluble proteins (Table 2).

Proteins are divided into two primary classes in the DeepTMHMM output: soluble and transmembrane. Proteins that are categorized as soluble are anticipated to perform their intended functions in the cell cytoplasm or other watery compartments. In contrast, transmembrane proteins feature one or more transmembrane helices and are most likely encapsulated within cellular membranes. Based on the outputs from the above-mentioned predictions (signal peptide, secretory protein, trans-membrane helix), the given proteins were found to have the following combinations of properties (given the fact that all are membrane-bound or periplasmic).

(1) Signal Peptide- Present; Secretory Protein- Yes; Trans-membrane Helix- No

It suggests that the protein is probably intended for secretion via the secretory route. Although it lacks parts that bridge cellular membranes, it is anticipated to be discharged from the cell or directed to extracellular compartments. These proteins (**WP_010359741.1, WP_003690747.1, WP_010951360.1**) may play a role in extracellular processes or are linked to particular secretory organelles within the cell.

(2) Signal Peptide- Absent; Secretory Protein- Yes; Trans-membrane Helix- Yes

This implies that the protein is projected to lack a signal peptide, be categorized as a secretory protein, and possess transmembrane helices, it may also follow non-classical secretion pathways and be connected to cellular membranes. It probably serves as a membrane protein, taking part in membrane-related processes and maybe releasing the signaling molecules, growth factors, or cellular components during processes like exosome release or cell shedding. Following hypothetical proteins belong to this category- **WP_010951062.1, WP_010951199.1, WP_003705520.1 and WP_010951229.1**.

(3) Signal Peptide- Absent; Secretory Protein- No; Trans-membrane Helix- Yes

This type of protein (**WP_232469644.1**) is anticipated to be an integral membrane protein if it lacks a signal peptide, is categorized as a non-secretory protein, and has transmembrane helices. This indicates that the protein is incorporated into cellular membranes and probably performs its function there. It could play a role in structural support, signal transduction, or membrane transport mechanisms. Its major function is probably within the cell and not in extracellular compartments because it is not anticipated to be secreted.

(4) Signal Peptide- Present; Secretory Protein- No; Trans-membrane Helix- No

This protein (**WP_010357457.1**) is likely targeted to a particular cellular compartment other than the secretory route if it is anticipated to have a signal peptide, be categorized as a non-secretory protein, and have no transmembrane helices. It is neither engaged in secretion or membrane-related processes nor does it bridge the cellular membrane.

As a result, if a protein is anticipated to have a signal peptide and is positive in SecretomeP but is identified by DeepTMHMM as a soluble protein, the protein will likely be secreted despite lacking transmembrane sections. This shows that rather than being entrenched within cellular membranes, the protein is targeted for secretion and may participate in extracellular processes. Out of the initial twenty proteins that were shortlisted, one protein (**WP_003690500.1**) was discovered to have extremely odd predicted features. It was not classified as secretary and was projected to have a trans-membrane while being cytoplasmic and lacking a signal peptide. The following explanations might be possible for this unusual behavior-

(i) Dual Localization: The protein might have dual localization, which would suggest that it is present in both the cytoplasm and near membranes. This could be a result of the protein having various iso-forms that allow for varied localizations within the cell.
(ii) Despite the possibility that the protein is not secreted, it may interact with intracellular membranes, including those of the endoplasmic reticulum, the Golgi apparatus, or other organelles. Transmembrane regions that aid the protein’s connection with particular membranes may be involved in these interactions.
(iii) Non-canonical Transmembrane Region: The anticipated transmembrane region might have special qualities or traits that differ from those seen in other membrane-spanning sections. Some proteins have been found to have unusual transmembrane topologies or alternate membrane connection mechanisms.

We selected **four** proteins (**WP_010951062.1, WP_010951199.1, WP_010951229.1, WP_010951360.1**) for further analysis based on following criteria:

Protein ismembrane-associated (Inner, outer, or periplasmic) and has at least one trans-membrane helix (as predicted by DeepTMHMM or HMMTOP) regardless of having a signal peptide. All these proteins were also predicted positively by SecretomeP 2.0. All these four proteins were then subjected to different web servers such as VirulentPred, Vaxijen 2.0, AllerTOP v2.0 to predict the virulence, antigenicity, allergenicity respectively (Table 3).

**TABLE 3:** Antigenicity, virulence, and allergenicity prediction output of four shortlisted proteins.

S. No.	Protein accession ID	VirulentPred	Vaxijen 2.0 score	AllerTOP v2.0
1	WP_010951062.1	Non-virulent	0.7161 (Probable Antigen)	Non-allergen
2	WP_010951199.1	Non-virulent	0.3845 (Probable Non-antigen)	Non-allergen
3	WP_010951229.1	Non-virulent	0.3942 (Probable Non-antigen)	Non-allergen
4	WP_010951360.1	Non-virulent	0.6790 (Probable Antigen)	Non-allergen

As can be observed from Table 3, all these proteins were predicted to be non-virulent and non-allergenic with varying degrees of antigenicity. It is important to mention that choosing epitopes or antigens that are immunogenic, non-virulent (risk-free, even for immune compromised individuals), and capable of offering protection without causing harm is a crucial step in the development of vaccines. The goal is to maximize the potential for beneficial effects while inducing a specific immune response against the pathogen or disease target. Based on these criteria, only two proteins (**WP_010951062.1, WP_010951360.1**) were selected as they were non-virulent, non-allergenic, and probable antigenic (Vaxijen score >0.5) as potential vaccine targets. From here onwards we will refer to them as **protein_1**(**WP_010951062.1),** and **protein_2** (**WP_010951360.1)** respectively and use for epitope prediction.

### CTL epitope prediction

The final shortlisted highly antigenic proteins were then used to predict CTL (cytotoxic T lymphocyte) epitopes, T-helper epitopes, and linear and continuous B-cell epitopes described as further. We used Net CTLpan v 1.1 server for 12 HLA supertypes (A1, A2, A3, A24, A26, B7, B8, B27, B39, B44, B58, and B62) to predict possible CTL epitopes. Due to their combined scores being greater than 1.5, four epitopes from **protein_1**and thirteen epitopes from **protein_2** were chosen as shown in Supplementary Table S3.

The relevant HLA allele and IC_50_ values were then predicted using the SMM approach implemented in the IEDB MHC-I epitope prediction tool. We selected only those MHC-I alleles that have an IC_50_ value of less than 250 nM to interact with the epitopes (Methodology Section). We obtained a total of 15 epitopes from these two proteins, 4 from protein_1 and 11 from protein_2 (Supplementary Table S3). Peptides SVVRGYFGY, LVIAVIASM, KQYAGKLGKfromprotein_2interacted each with maximum five different HLA alleles. Furthermore, using the “IEDB Class I Immunogenicity tool” with default settings, immunogenic peptides from these CTL epitopes restricted to MHC-I were also predicted (Supplementary Table S4).

Eight of these fifteen CTL epitopes were predicted to be immunogenic, while seven were discovered to be non-immunogenic. Antigenicity predictions for these immunogenic epitopes revealed that only two peptides (GSIEGMEQY and EEIPFDLYL) are antigenic. According to their Vaxijen scores, the former peptide was found to be weakly immunogenic while the latter was found to be highly immunogenic. The results are shown in Supplementary Table S5.

**FIGURE 2:**
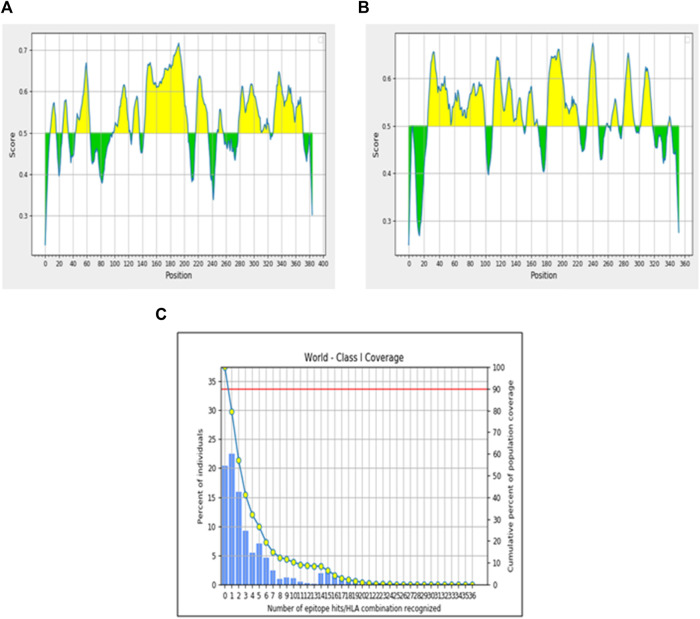
Population coverage analysis of immunogenic epitope. **(A)** Linear B-cell epitope scores for Protein 1. **(B)** Linear B-cell epitope scores for Protein 2. **(C)** Population coverage by predicted T-cell epitopes.

Toxicity analysis of these two epitopes by ToxinPred revealed that both the epitopes were predicted to be non-toxic, while the allergenicity assessment by AllerTOP v2.0 showed that only EEIPFDLYL was predicted to be non-allergenic while GSIEGMEQY was found to be allergenic. Our final shortlisted CTL epitope, EEIPFDLYL was predicted to be restricted by four HLA alleles (HLA-C*03:03; HLA-B*40:01; HLA-B*15:02; HLA-B*44:03) as shown in Supplementary Table S3. Population coverage analysis was also carried out for this immunogenic CTL epitope (**EEIPFDLYL**) to comprehend the immune responses in various populations. This thorough portrayal enhances our understanding of immunology and the production of vaccines globally and enables a more inclusive and accurate understanding of immune responses. About 22.92% of the world’s population is represented by the chosen epitope. East Asia (39.05%) has the highest population coverage, followed by Northeast Asia (34.86%), North America (24.66%), Europe (21.98%), South Asia (10.85%), and South America (10.74%) Figure 2. The results are shown in Table 4.

**TABLE 4:** Population coverage by the selected epitope using IEDB.

Population/Area	Coverage (%)	Average hit	PC90^a^
World	22.92	0.25	0.13
Europe	21.98	0.24	0.13
East Asia	39.05	0.45	0.16
Northeast Asia	34.86	0.39	0.15
South Asia	10.85	0.11	0.11
North America	24.66	0.27	0.13
South America	10.74	0.11	0.11

^a^ Number of epitopes required to elicit an immune response in 90% of the population.

### CD4^+^ T-cell epitope prediction

#### MHC-II binders prediction

The IEDB MHC-II binding prediction approach was used to predict MHC-II binders from protein_1 and protein_2 sequences. We utilized peptides with IC_50_ values smaller than 50 nM as a cut-off to choose strong binders as per IEDB recommendations (Wang et al., 2008). We selected the top one percentile sequences as very strong binders. According to these criteria, sixteen strong binders were obtained for protein_1 (Supplementary Table S6).

The “CD4 T cell immunogenicity prediction tool” available at the IEDB predicted only one peptide (**ESEIAALKAVLAKAD**) as immunogenic among all the strong MHC-II binders from protein_1. It was found to have a combined score of 46.82 and an immunogenicity score of 91.57 for seven MHC-II alleles as shown in Supplementary Table S7.

This peptide was found to be antigenic (Vaxijen score = 0.4028), non-allergenic and non-toxin as predicted by AllerTOP v2.0 and ToxinPred respectively. Similarly, MHC-II binders were predicted for protein_2 which resulted in twenty eight strong binders (top one percentile). The result is shown in Supplementary Table S8.

Among all these twenty-eight strong MHC-II binders, none of the peptides were immunogenic using the “CD4 T cell immunogenicity prediction tool” (at default threshold) available at the IEDB. Hence, these peptides were not evaluated for antigenicity, toxicity, or allergenicity.

### B-cell epitope prediction

#### Linear B-cell epitope prediction

**FIGURE 3:**
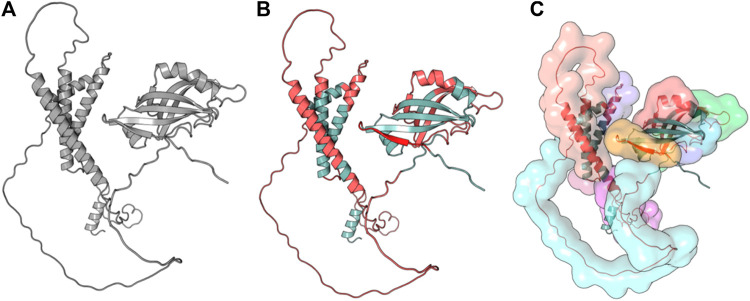
Full-length conformation of Protein 1 (WP_010951062). **(A)** Native structure from UniProt (Q5F9A2). **(B)** Linear B-cell epitopes in red, rest in gray. **(C)** Surface view of predicted epitope regions (colored).

**FIGURE 4:**
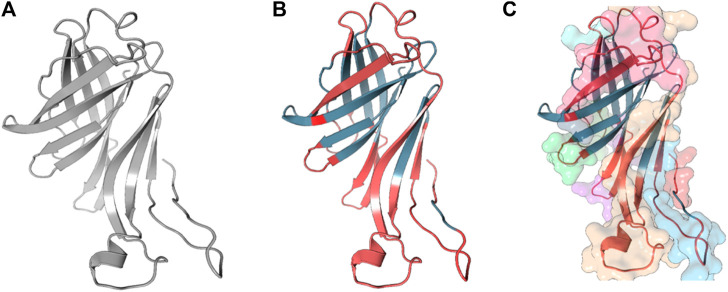
Full-length conformation of Protein 2 (WP_010951360). **(A)** Native structure predicted using Phyre2. **(B)** Linear B-cell epitopes in red, rest in gray. **(C)** Surface view of predicted epitope regions (colored).

Linear B-cell epitopes were predicted using the “Antibody epitope prediction” module of IEDB with default setting (Bepipred linear epitope prediction 2.0) for protein_1 and protein_2. The length of predicted epitopes of protein_1 ranged from 9 to 63 amino acid residues. The length of predicted linear B-cell epitopes of protein_2 ranged from 2 to 75 amino acid residues. The results are shown in Tables 5, 6 respectively and Figures 3, 4 respectively.

**TABLE 5:** Bepipred 2.0 linear B-cell epitope prediction results for protein-1(WP_010951062.1).

S. No.	Start	End	Peptide	Length
1	8	16	IAKTEAQDD	9
2	26	34	SSEAVDSDG	9
3	45	65	AIPDYMKFGAVREMHGSNAAG	21
4	98	123	YKGFSIGGSVTARNDLNKSQITGLKL	26
5	127	136	SLVDRPANPD	10
6	144	206	ADKPKDEAGAADKDGKPSDKPTEEEDENPKDGDKGPKTEDKGDKDAGKKDEAGKSASVNLSES	63
7	218	235	ADKPKGGPAAKSMYQVKS	18
8	248	257	EDASYDNIDE	10
9	279	324	ASEADKPADGLAAKAGKSGDLAKAESADELAKAQDALKKSNDALAK	46
10	329	371	IESLKKQAVPPKGSTKAISKAEDNGEDPLKGFQPIVKNDGTLD	43

**TABLE 6:** Bepipred 2.0 linear B-cell epitope prediction results for protein_2(WP_010951360.1).

S. No.	Start	End	Peptide	Length
1	26	100	GGGSDSSMSVQPSVSEQLKDNANVDAKDEKVIEYLKKSSLDVPKELQAKVLKVKGDEYTGVRKQYAGKLGKGES	75
2	111	150	EPFSKEQLQKMDVYVNGKKYEGSKGGELDVLPKGLSEQKI	40
3	154	169	GADKEQNYALLKTWVY	16
4	181	222	GYSRKDGNPIEGDGQNPEEIPFDLYLGDIRGVATDEDKLPKA	42
5	233	248	GGNGVLSKESLDNHNG	16
6	259	260	RK	2
7	265	274	IEGMEQYGKI	10
8	280	295	AIERIPYRESGSSLGL	16
9	304	318	VNEGVAMLEKDNEIK	15
10	339	343	EHKHQ	5

Further analysis of these twenty linear B-cell epitopes from both the proteins for antigenicity showed that thirteen peptides were antigenic, seven peptides from protein_1, and six peptides from protein_2 as per Vaxijen v2.0 prediction (Supplementary Table S9). Two peptides from protein_2 had a sequence length of less than nine, hence their antigenicity cannot be predicted.

Toxicity and allergenicity of these thirteen antigenic peptide sequences showed that five (out of seven) sequences from protein_1 were non-toxic and non-allergenic, while only two (out of six) were non-toxic and non-allergenic from protein 2 (Table 7 **)**.

**TABLE 7:** Antigenic, non-toxic, and non-allergenic linear B-cell epitopes from protein_1 and protein_2.

Protein_1	
S. No.	Antigenic, non-toxic and non-allergenic linear B-cell epitopes
1	SSEAVDSDG
2	AIPDYMKFGAVREMHGSNAAG
3	YKGFSIGGSVTARNDLNKSQITGLKL
4	ADKPKGGPAAKSMYQVKS
5	ASEADKPADGLAAKAGKSGDLAKAESADELAKAQDALKKSNDALAK

Protein_2	
S. No.	Antigenic, non-toxic and non-allergenic linear B-cell epitopes
1	EPFSKEQLQKMDVYVNGKKYEGSKGGELDVLPKGLSEQKI
2	GYSRKDGNPIEGDGQNPEEIPFDLYLGDIRGVATDEDKLPKA

### Prediction of conformational (discontinuous) B-cell epitopes

The majority of B cell epitopes, contrary to conventional belief, are discontinuous or conformational (Novotný et al., 1986). The 3D structures of the proteins (protein_1 and protein_2) were developed and uploaded to the IEDB-integrated Ellipro method to predict discontinuous B cell epitopes. Protein_1 was found to have four conformational epitopes, while protein_2 had three (Table 8).

**TABLE 8:** Conformational B-cell epitopes predicted for protein_1 and protein_2 using Ellipro tool.

Protein_1			
S. No.	Residues	No. of residues	Score
1	_:E248, _:D249, _:A250, _:S251, _:Y252	5	0.965
2	_:D253, _:N254, _:I255, _:D256, _:E257	5	0.829
3	_:E272, _:K275, _:A276, _:A278, _:A279, _:S280, _:E281, _:A282, _:D283, _:K284, _:P285, _:A286, _:D287, _:G288, _:A290, _:A291, _:A293, _:G294, _:K295, _:S296, _:G297, _:D298, _:L299, _:A300, _:K301, _:A302, _:E303, _:S304	28	0.659
4	_:Q312, _:D313, _:A314, _:L315, _:K316, _:K317, _:S318, _:N319	8	0.594

Protein_2			
S. No.	Residues	No. of residues	Score
1	B:T166, B:W167, B:E170, B:Q171, B:N196	5	0.646
2	B:Y179, B:G181, B:Y182, B:S183, B:R184	5	0.583
3	B:G192, B:D193, B:G194	3	0.524

**FIGURE 5:**
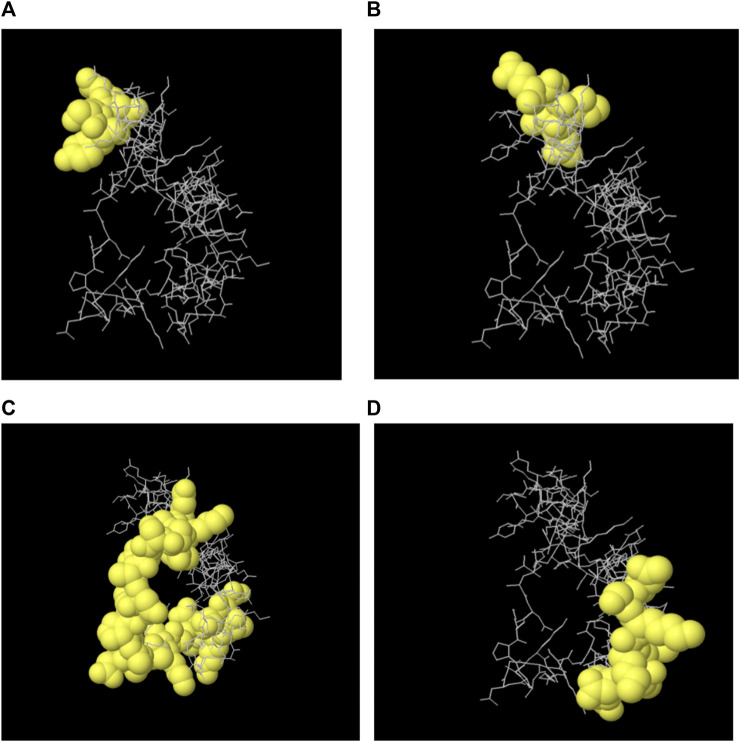
Conformation of the four B-cell epitopes predicted for Protein 1 using Ellipro method. **(A–D)** Four epitopes: yellow balls represent the relevant epitope residues and white sticks indicate the structure of core residues.

**FIGURE 6:**
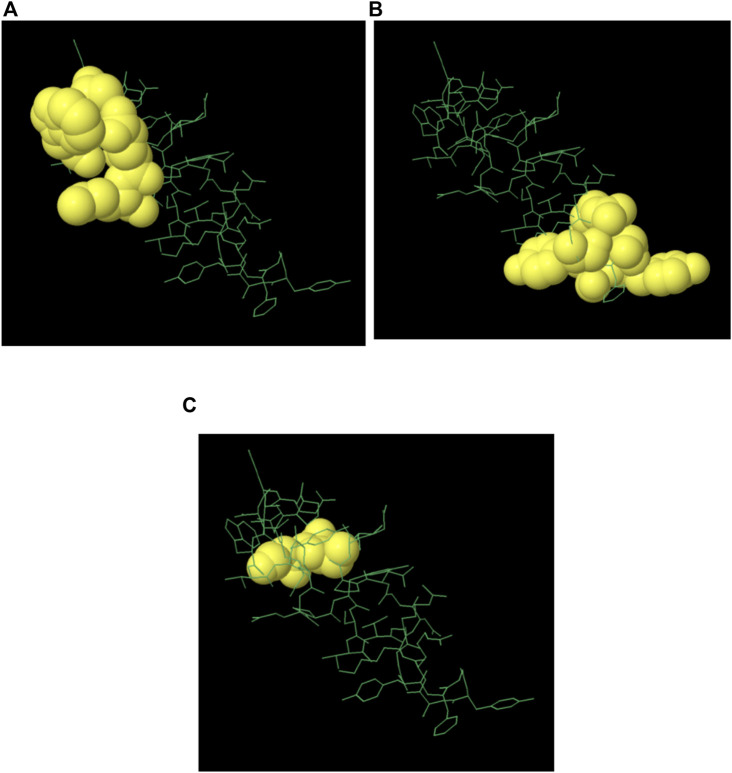
Conformation of B-cell epitopes of Protein 2 as predicted by Ellipro method. **(A–C)** Three epitopes: yellow balls represent the relevant epitope residues and white sticks indicate the structure of core residues.

**FIGURE 7:**
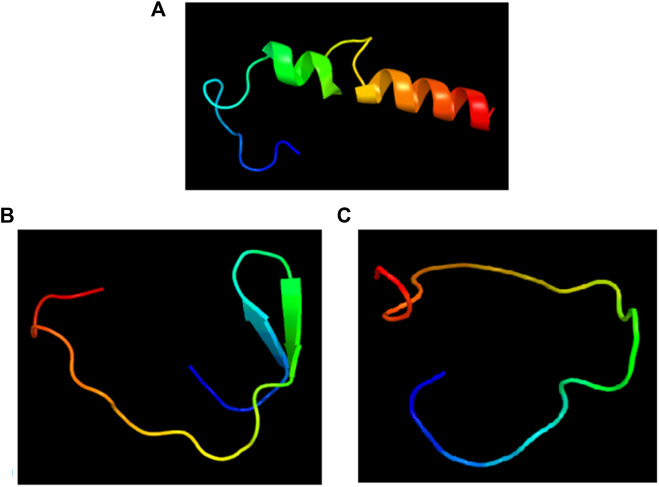
Structures of the linear B-cell epitope predicted to showcase the epitope regions from Protein 1.**(A)** and Protein 2 **(B,C)**.

The 3D structures of the epitopes, which show their specific locations inside the protein, were visualized using Jmol (integrated with Elipro module at IEDB). The full-length sequences of both proteins were used to predict the epitope residues, which were dispersed across the surface. The prediction parameters of the Ellipro method were a minimum score of 0.5 and a maximum distance of 6 Angstrom (Å). The epitope scores range from 0.594 to 0.965 for protein_1 and 0.583 to 0.646 for epitope_2. Figures 5A–D, 6A–C shows a detailed view of these conformational epitopes for protein_1 and protein_2 respectively. We have further predicted the structures of the proposed linear B-Cell epitopes using Phyre2 server as shown in Figures 7A–C.

## Discussion

The quest for alternative vaccines against *N. gonorrhoeae* stems from the surging global prevalence of the disease, the rise of antibiotic-resistant strains, and the complexities in treatment. Our study endeavours to address this pressing need by introducing an innovative *in silico* methodology, amalgamating functional annotation of hypothetical proteins (HPs), and immuno-informatics predictions to identify potential vaccine candidates.

A substantial fraction of *N. gonorrhoeae* FA 1090 strain’s proteome (around 800 polypeptides) comprises proteins with unknown functions and biochemical characteristics. Using artificial intelligence and machine learning-driven tools we tried tounravel the structure and function of these HPs. Leveraging these methodologies, we have successfully forecasted various attributes including functional annotations, structural features, physicochemical properties, sub-cellular localizations, antigenicity, and the presence of virulence factors for these proteins. Additionally, we have endeavoured to comprehend the putative functions and biological significance of *N. gonorrhoeae* HPs in the context of pathogenicity and infection development. To ascertain the sub-cellular localization of the identified proteins, we adopted a consensus-based approach by integrating multiple methodologies, each contributing its distinct advantages and algorithms to enhance predictability and accuracy.

Building on Mondol et al.'s work on *R. capsulatus* (Mondol et al., 2022) and similar studies in *S. coelicolor* (Ferdous et al., 2020), our research expands understanding of *N. gonorrhoeae* by identifying diverse functional hypothetical proteins (HPs). Notably, we identified a subset of HPs exhibiting oxidoreductase activity, crucial for electron transfer reactions vital for bacterial metabolism and energy production. Furthermore, our analysis revealed a proportion of annotated HPs as proteases, implying potential roles in various cellular processes, including nutrient breakdown, protein turnover, and possibly virulence factor activation.

Studying a hypothetical protein in *C. trachomatis*-infected cells (Li et al., 2011) and similar proteins in *Haemophilus influenzae* Rd KW20 (Shahbaaz et al., 2013) highlights their roles in inclusion membrane formation and pathogenesis, emphasizing the significance of understanding bacterial hypothetical proteins in research and therapeutic targeting. In line with these findings, our study identified a subset of HPs predicted to possess transmembrane helices, suggesting their involvement in membrane transport, signal transduction, and receptor activation. Additionally, studying putative hypothetical proteins from *Candida dubliniensis* demonstrates the effectiveness of bioinformatics tools in pinpointing specific functions, advancing our understanding of pathogenesis and aiding drug discovery (Kumar et al., 2014). Our characterization of a subset of these proteins as Synthases/Hydrolases suggests their involvement in synthesizing and breaking down molecules, potentially crucial for cell wall maintenance and metabolite production.

The development of effective vaccines against pathogens necessitates a comprehensive understanding of their virulence factors, protein functionalities, and mechanisms of immune evasion. This knowledge forms the cornerstone for identifying suitable targets for vaccine design and ensuring the elicitation of robust immune responses. Central to our study was the quest to identify optimal HP-derived subunit epitope-based vaccine candidates. We therefore, employed an *in silico* approach to identify potential vaccine candidates against *N. gonorrhoeae* by integrating functional annotation of hypothetical proteins (HPs) and immuno-informatics prediction of epitope-based peptide vaccines. Epitopes within proteins serve as key sites capable of triggering immunological responses. Immuno-informatics explores immune and epitope relationships, developing tools for antigen response prediction (Patronov and Doytchinova, 2013; Sanchez-Trincado et al., 2017). Thus, *in silico* studies can play a crucial role in predicting epitopes capable of eliciting both T-cell and B-cell responses, thereby fostering cellular and humoral immune responses. Using these tools, we prioritized B cell and T cell epitopes within the HPs, to identify those with the potential to yield effective vaccines against *N. gonorrhoeae*. A similar immuno-informatics strategy was used recently to develop vaccines against SARS-COV2 by Feng et al. (Zhang et al., 2023).

Our study predicts *N. gonorrhoeae* protein epitopes, aiding vaccine development against this sexually transmitted disease. Notably, the induction of a potent immunological response, particularly one driven by B cells, is pivotal for vaccine success. Identification of linear B-cell epitopes within the proteins of interest strongly suggest its potential to be a good vaccine candidate as these epitopes are recognized by antibodies produced by B cells (Potocnakova et al., 2016).

Helper T-lymphocytes (HTLs) are crucial for triggering cellular and humoral immune reactions, emphasizing the importance of epitopes recognized by these cells in preventative and therapeutic vaccinations. Our study predicts the multiple B-cell and T-cell epitopes within HPs of *Neisseria gonorrhoeae* supporting the rational design of multiepitope vaccines against this antibiotic-resistant pathogen. Ahmad et al. (2019) earlier used a multiepitope strategy to design vaccines to combat tigecycline resistant *Acinetobacter baumannii.* (Ahmad et al., 2019; Majidiani et al., 2021), predicted major histocompatibility complex (MHC)-binding and B-cell binding epitopes of five Toxoplasma antigens. Selected epitopes were fused and checked for secondary and tertiary structures, allergenicity, physicochemical features, and antigenicity using *in silico* tools and experimentally validated for efficacy using BALB/c mice. The recombinant, multi-epitope vaccine when expressed in *Leishmania tarentolae* induced significant immune responses against acute toxoplasmosis (Majidiani et al.) (Majidiani et al., 2021). Universal multi-epitope vaccine involving three highly immunogenic proteins of *Streptococcus suis* was designed using the immuno-informatics approach (Segura, 2015; Jalal et al., 2023; Shafaghi et al., 2023; Waqas et al., 2023; Zhang et al., 2023; Liang et al., 2024). Similarly, multiepitope vaccines were predicted against different strains of *Streptococcus pneumoniae* (Shafaghi et al., 2023). Comparing our findings with studies on diverse organisms informs vaccine development strategies, revealing commonalities and unique features. Computational methods like structural prediction and epitope mapping efficiently screen pathogen proteins, identifying antigenic regions for vaccine optimization.

Our study highlights the pivotal role of computational methodologies in both functional annotation and vaccine design processes. By delving into the intricate realm of *N. gonorrhoeae* hypothetical proteins, we have not only expanded our understanding but also showcased the efficacy of *in silico* methodologies in unravelling their functional roles. Furthermore, through the precise utilization of bioinformatics tools, we have pinpointed promising vaccine candidates for *N. gonorrhoeae*, marking a significant stride in global efforts to effectively control and eradicate this sexually transmitted disease.

## Limitations of the study

Detailed analysis of all hypothetical proteins (HPs) led to the confident functional annotation of twenty proteins. Further investigation is however, necessary to ascertain the precise functions of these GDC proteins. Their diverse repertoire hints at their potential involvement in multiple facets of *N. gonorrhoeae* biology and pathogenesis. Unravelling these functions promises valuable insights into the mechanisms underlying *N. gonorrhoeae* infection, potentially paving the way for innovative therapeutic approaches. We have also successfully identified promising *N. gonorrhoeae* vaccine candidates through further refinement of our approach. However, it is crucial to note that *in silico* predictions, although beneficial, can be prone to errors, emphasizing the need for experimental validation to substantiate our findings. Moreover, *N. gonorrhoeae*, being an intracellular pathogen, may evade immune responses targeted at these antigens. Therefore, it is imperative to validate our current approach both *in vitro* and in animal models to assess its effectiveness comprehensively.

## Materials and methods

Our study aims to offer thorough insights into the functional and structural aspects of *N. gonorrhoeae* HPs by combining several bioinformatics techniques. Additionally, to develop focused and successful methods for battling *N. gonorrhoeae* infections, our research aimed to find new vaccine candidates by anticipating and characterizing the epitopes within these HPs. Supplementary Table S6 depicts the entire framework and the tools used in this investigation. The entire procedure includes three phases: Phase I, Phase II, and Phase III.

**Phase I** involves genomic analysis and characterization of specific hypothetical proteins (HPs). The genomic data of the pathogenic organism, *N. gonorrhoeae* was scrutinized to pinpoint and categorize the HPs encoded within the genome. This process encompasses delineating their precise genomic loci, deciphering potential protein sequences, and identifying open reading frames (ORFs). Furthermore, available information pertinent to these HPs, such as putative functions or conserved domains, was investigated.

**Phase II** encompasses the utilization of multiple computational tools and diverse bioinformatics approaches to annotate and delineate the functional attributes of the HPs. This multifaceted analysis involves the assessment of their physicochemical characteristics, sub-cellular localizations, antigenic profiles, virulence factors, and other significant attributes. The integration of various computational tools broadens the scope of analysis, facilitating a comprehensive understanding of the functional landscape exhibited by the HPs.

**Phase III** revolves around the prioritization of potential targets conducive to vaccine development against the pathogen. Within the spectrum of HPs, candidates with the potential for vaccine development emerge based on the comprehensive analysis and annotation conducted in the earlier phases. These candidates undergo meticulous selection based on their predicted functional attributes and their suitability for vaccine formulation.

### Phase I

#### Sequence retrieval

To initiate our study, the complete genome sequence of *N. gonorrhoeae* strain ATCC 700825/FA 1090 was obtained from the NCBI database with GenBank assembly GCA_000006845.1 and RefSeqNC_002946.2 (UniProt, 2024). Initially, 890 Hypothetical Proteins (HPs) were identified within the genome. The protein sequences of these HPs were available on NCBI website and were retrieved from there. Upon implementation of the filtering procedure to reduce the redundancy and to refine the dataset, we obtained 824 distinct protein sequences. The extracted sequences (*n* = 824) were saved as FASTA files for subsequent analysis. CD-HITv4.7 was then utilized (at default setting) to effectively group and eliminate highly similar sequences (Fu et al., 2012). This resulted in a refined set of 632 protein sequences. To precisely understand the fundamental characteristics of these 632 protein sequences, we subjected them toa variety of bioinformatics tools (Supplementary Table S6) to predict properties such as protein functions, structures, physicochemical properties, sub-cellular localizations, antigenicity, and other significant features pertinent to their potential roles in the pathogenesis of *N. gonorrhoeae*.

#### Conserved domain exploration in hypothetical protein sequences

Proteins are composed of domains and functional units that execute specific tasks and may exhibit recurring patterns or distinctive structures. Employing bioinformatics tools such as CDD-BLAST (Marchler-Bauer et al., 2015), SMART (Letunic et al., 2021), PFAM (Finn et al., 2014), ScanProsite (de Castro et al., 2006) and InterProScan (Quevillon et al., 2005), we aimed to elucidate potential functional attributes embedded within these proteins. These computational tools scrutinize the amino acid sequences of HPs, aligning them with established protein databases to predict the conserved domains or structural folds, facilitating the classification of HPs into protein families, and understanding their role in biological processes. This acquired knowledge serves as a cornerstone for future investigations, shedding light on the intricate molecular relationships and mechanisms governing HPs. Moreover, the discernment of distinct and conserved domains, often indicative of pivotal functional roles, aids in prioritizing HPs warranting further in-depth scrutiny. The proteins predicted by all these five bioinformatics tools were retained for further analysis and are classified under the label “Great Degree of Confidence” “(GDC)” for this study.

### Phase-II

#### Physicochemical characterization

Several physicochemical features of the above HP’s with a great degree of confidence (GDC) were evaluated for a better understanding of their properties. Theoretical iso-electric point (pI), molecular weight (MW), total amino acid count, aliphatic index, and extinction coefficient were calculated by using the Expasy ProtParamserver (Gasteiger et al., 2003), a commonly used bioinformatics tool whereas the grand average of hydropathy (GRAVY) was calculated using GRAVY Calculator tool (Kyte and Doolittle, 1982). Total number of positively and negatively charged residues, and instability index are a few significant physicochemical properties that were determined. We also determined the iso-electric point (pI), which sheds light on a protein’s solubility and electrophoretic behavior, molecular weight (MW), which is a measure of the size of a protein, and the total number of amino acids which provides insight into the length and complexity of the protein as a whole. The aliphatic index measures the proportional volume occupied by aliphatic amino acids to provide light on the protein’s structural stability and thermo stability (Ikai, 1980). The protein’s light absorption properties are reflected by the extinction coefficient, which can be used in measurement and purification methods (Gill and von Hippel, 1989). The grand average of hydropathy (GRAVY) is a measure of a protein sequence’s hydrophobic or hydrophilic character (Kyte and Doolittle, 1982). It helps forecast its behavior in various biological processes and shows how it might interact with hydrophobic surroundings.

The overall charge distribution of the protein is also influenced by the sum of positively and negatively charged residues, which can have an impact on how the protein interacts with other molecules. Last but not least, the instability index (Guruprasad et al., 1990) estimates the protein’s stability; higher values denote a greater likelihood of disintegration.

We can learn more about the GDC protein’s structural features, solubility, stability, and potential interactions with biological systems by assessing their physicochemical qualities. These investigations help to fully characterize these proteins and shed light on how they function within the cell of *N. gonorrhoeae*.

#### Sub-cellular localization

Protein activities are typically connected to their sub-cellular location. Thus, the capacity to anticipate sub-cellular localization directly from protein sequences will be valuable for determining its cellular activities. Proteins that are located in the cytoplasm are well known to be possible drug targets, whereas proteins that are located on the surface of membranes are expected to be targets for vaccines (Jiang et al., 2021). Because the HPs have not been experimentally characterized, there is a knowledge gap and their sub-cellular localizations are obscured.

We used several bioinformatics tools known for their precision and dependability to ascertain the sub-cellular localization of the shortlisted proteins, namely, CELLO (v2.5) (Yu et al., 2004), CELLO2GO (Yu et al., 2014), PSORTb (Yu et al., 2010), and PSLpred (Bhasin et al., 2005), and others. A two-level Support Vector Machine (SVM) system is used by CELLO (v2.5) to predict sub-cellular localization. It gives information about the potential cellular organelles or compartments of proteins by analyzing their sequences. CELLO2GO uses functional gene ontology annotation to forecast sub-cellular localization (Yu et al., 2014). It assigns functional annotations and forecasts protein localization to certain biological components using the abundance of knowledge contained inside the Gene Ontology database. CELLO2GO attains a remarkable 99.1% accuracy in predicting sub-cellular localization for Gram-negative bacteria, 99.4% for Gram-positive bacteria, and 98.4% for archaeal sequences (Yu et al., 2014) PSORTb stands out as the most precise localization prediction tool, boasting an accuracy of 96% for both Gram-negative and Gram-positive bacteria (Yu et al., 2010). CELLO maintains a high prediction accuracy of 89%, ensuring precision and reliability in its predictions. Based on the previous reports and prediction accuracy, PSORTb is one of the most widely and effective sub-cellular localization prediction tools (Yu et al., 2010). To correctly predict the sub-cellular localization of bacterial proteins, a variety of sequence characteristics and signal peptides are taken into consideration. Another technique for predicting sub-cellular localization is PSLpred (Bhasin et al., 2005). For precise predictions, it uses a hybrid technique based on PSI-BLAST and three SVM modules, taking into account residue compositions, di-peptides, and physicochemical features. PSLpred achieves an overall accuracy of 89% for prokaryotic protein localization. It should be noted that PSLpred places a strong emphasis on foretelling sub-cellular localization in Gram-negative bacteria. In addition, we employed the neural network-based system SignalP 6.0 (Emanuelsson et al., 2007), SecretomeP2.0 (Bendtsen et al., 2004) to forecast signal peptides and secretory pathways (non-classical). SOSUI (Hirokawa et al., 1998), TMHMM (Krogh et al., 2001), DeepTMHMM (Hallgren et al., 2022), CCTOP (Dobson et al., 2015), TOPCONS (Tsirigos et al., 2015)and HMMTOP (Tusnády and Simon, 2001) were also employed in our work to predict transmembrane structure and protein solubility.

#### Function prediction

We used multiple servers to accurately predict the protein’s specific roles. The domains were searched using CDD (Conserved Domain Database) (Wang et al., 2023), ScanProsite (de Castro et al., 2006), SMART (Letunic et al., 2021), Pfam (Finn et al., 2014) and InterProScan (Quevillon et al., 2005) was also utilized; it employs the InterPro consortium and its several databases, including Pfam, SUPERFAMILY, SMART, PANTHER (Thomas et al., 2022), and ProSite, to perform a mix of protein signature recognition methods.

#### Protein structure prediction

Two final shortlisted proteins, WP_010951062 and WP_010951360, were subjected to protein structure prediction analysis as part of our methodology. The structure of WP_010951062, consisting of 385 amino acid residues, was retrieved from the UniProt database with the accession number Q5F9A2. The UniProt database provides curated protein sequence information, including experimentally determined structures, annotations, and functional details for proteins of various organisms. Concomitantly, the structure of WP_010951360, comprising 353 amino acid residues, was predicted using Phyre2, an established web-based tool for protein structure prediction (Kelley et al., 2015). Phyre2 employs advanced algorithms incorporating homology modeling, *ab initio* folding, and threading methods to generate reliable 3D structure predictions based on amino acid sequences. The selection of Phyre2 for the prediction of the structure of WP_010951360 was based on its ability to provide accurate and detailed structural models, guiding our exploration of the protein’s potential conformation and functional implications.

#### Virulence factor prediction

Drug development focuses on virulence factors (VFs), which are connected to the strength or severity of an infection. Understanding the intricate virulence process of pathogenesis and determining a bacterium’s pathogenic potential benefit from the identification of virulent proteins in its protein sequences. Two bioinformatics methods were used to identify the virulence factors (VFs) of the chosen hypothetical proteins (HPs): VirulentPred (Sharma et al., 2023)and VICMpred (Saha and Raghava, 2006). In this investigation, both VirulentPred, with an accuracy of 81.8%, and VICMpred (Saha and Raghava, 2006), with an accuracy of 70.75%, were used.

A potent machine learning method, Support Vector Machine (SVM) technology is used by both VirulentPred and VICMpred to forecast the existence of VFs in the protein sequences. Both servers use a five-fold cross-validation procedure to guarantee accurate predictions. By splitting the dataset into five subsets, training the model on four of them, and validating it on the fifth, this validation technique aids in evaluating the performance of the prediction models. Each subset serves as the validation set once throughout each of the subsequent five iterations of this process. Hence, using extensive datasets for development and validation, VirulentPred and VICMpred can provide precise predictions about the existence of VFs in the HPs. These techniques were used in this study to uncover possible virulence factors within the GDCHPs and provide insight into their potential roles in *N. gonorrhoeae* pathogenicity and disease progression.

#### Prediction of allergenicity

The potential vaccine candidate must not be allergic to the host to prevent the body from mounting an auto-immune response. For this, we used AllerTop v2.0 (Dimitrov et al., 2013) and AllerCatPro (Nguyen et al., 2022) to determine whether the protein would act as an allergen or non-allergen. The allergenic proteins were removed from the dataset for further analysis.

#### Prediction of antigenicity

We used VaxiJen v2.0 (Doytchinova and Flower, 2007) to assess the protein’s protective antigen potential. VaxiJen predicts antigenicity from protein sequences. This work defined a bacteria-specific threshold of 0.4 to identify putative protective antigens with high precision. We assessed the protein’s antigenicity and vaccination potential using the VaxiJen server. Proteins with higher VaxiJen scores are more immune-stimulating and protective. We chose the protein with the greatest antigenic score to predict B and T cell epitopes. This protein was prioritized because its sections are likely to trigger a significant immunological response and serve as vaccine targets. Both T cells and antibodies recognize these epitopes, which are critical to immunological responses. We used VaxiJen and selected the most antigenic protein to identify vaccine candidates that would induce an immune response and protect against *N. gonorrhoeae*. This method helps produce pathogen-specific vaccinations.

### Phase III

#### Prediction of linear and conformational B-cell epitopes

We used the “BepiPred Linear Epitope Prediction 2.0” approach, which is accessible through the B-cell epitope prediction tool offered by the Immune Epitope Database (IEDB) (Jespersen et al., 2017), to predict linear B-cell epitopes. The amino acid sequences from both non-epitopes and epitopes discovered in antigen-antibody crystal structures were used to train this tool. Based on the input protein sequences, it uses the Random Forest (RF) approach, a machine learning algorithm, to produce predictions (Jespersen et al., 2017). When making a prediction, amino acid residues that earn scores greater than the default threshold value of 0.5 are considered epitopes (Jespersen et al., 2017). The protein sequence is divided into areas that can likely act as B-cell epitopes using this threshold.

According to Sanchez-Trincado et al. (Sanchez-Trincado et al., 2017), conformational B-cell epitopes are made up of scattered or interrupted amino acid sequences within an antigen that interact with B-cell receptors (BCRs). Conformational epitopes, in contrast to linear epitopes, require the proper three-dimensional (3D) structure of the antigen for BCR recognition. We used the ElliPro tool from the Immune Epitope Database (IEDB) (Ponomarenko et al., 2008) to predict these discontinuous B-cell epitopes. Based on the protein antigen’s 3D structure, ElliPro uses a computer technique to anticipate conformational B-cell epitopes using default parameter values of 0.5 for the lowest score and 6 Angstrom (Å) for the greatest distance (Ponomarenko et al., 2008). The output provides information such as the number of residues, the score given to the epitope, the amino acid residues involved in the epitope, and a link to the 3D structure of the protein antigen (Ponomarenko et al., 2008). Using the Phyre2 protein structure prediction program (Kelley et al., 2015) the 3D structure of the protein was predicted and verified in this study. This stage was crucial to guarantee the precision and dependability of ElliPro’s predictions for locating conformational B-cell epitopes inside the protein antigen. We used ElliPro to examine the 3D structure to find potential discontinuous B-cell epitopes, which will help us better, understand the antigenic portions of the protein and develop *N. gonorrhoeae* vaccines.

#### Prediction of CD8^+^ T-cell epitopes

In the present study, the CD8^+^ T-cell epitopes specific to twelve HLA supertypes (A1, A2, A3, A24, A26, B7, B8, B27, B39, B44, B58, and B62) were predicted using the NetCTLpan v1.1 Server (Stranzl et al., 2010). This server accurately predicts Cytotoxic T- Lymphocytes (CTL) epitopes by using sequence-processing methods including proteasome cleavage, TAP binding, and MHC-I binding (Stranzl et al., 2010). The Immune Epitope Database (IEDB)’s MHC-I binding prediction tool was used to identify the corresponding HLA allele and the accompanying IC_50_ values for peptides with a combined score greater than 1.5. Epitopes with IC_50_ values under 250 nM were taken into consideration for a more thorough evaluation of immunogenicity (Anand et al., 2020; Jagadeb et al., 2021).

To find MHC-I-restricted immunogenic peptides, we used the “IEDB Class I Immunogenicity tool” with default settings (Calis et al., 2013). This tool generates scores by taking into account the characteristics of the amino acids and their placements in the sequence; higher scores denote a higher likelihood of eliciting an immunological response (Calis et al., 2013).

#### Prediction of CD4^+^ T-cell epitopes

We used IEDB MHC-II binding tool (Andreatta et al., 2018)to predict fifteen amino acid-long CD4^+^ T-cell epitopes. The “CD4 T cell immunogenicity prediction tool” available at the IEDB was used to predict the immunogenicity of MHC-II restricted peptides. The “IEDB recommended” technique, which combines the immunogenicity method with MHC-binding to seven alleles, was used to make the prediction (Dhanda et al., 2018).

#### Characterization of chosen B-Cell and T-Cell epitopes

The B-cell and T-cell predicted epitopes with significant cutoffs were examined for key characteristics such as antigenicity, toxicity, and allergenicity. The potential peptide-based vaccination epitopes need to be non-allergen, non-toxic, and antigenic.

#### Antigenicity, toxicity and allergenicity prediction

The antigenicity of the epitopes was predicted using the Vaxijen v2.0 web-server. The ToxinPred web server (Gupta et al., 2013) was used to forecast the toxicity of antigenic B-cell and T-cell epitopes with a Vaxijen score above 0.4 (Gupta et al., 2013). To avoid allergic reactions in the host by vaccination, potential vaccine candidates must also be tested for allergenicity (McKeever et al., 2004). We predicted the allergenicity of these predicted epitopes using AllerTOP v. 2.0that utilizes the k-nearest neighbors (kNN) method to distinguish between allergens and non-allergens (Dimitrov et al., 2013).

#### Population coverage prediction

The frequency of the various polymorphic HLAs varies among populations, and the epitopes that these HLAs restrict would have biased population coverage (Bui et al., 2006). To prevent a decrease in the applicability of a vaccine candidate in specific communities, population coverage must be taken into account while designing a vaccine (Bui et al., 2006). As a result, it is crucial to determine the proportion of people who are predicted to respond to a specific epitope set based on HLA type (Bui et al., 2006). The population coverage tool from the IEDB was used to determine the coverage of our proposed epitopes with the associated HLAs among the various ethnic groups.

## Conclusion

Our study uses an *in silico* methodology harnessing Artificial Intelligence and Machine Learning based tools to functionally annotate 632 hypothetical proteins of *N. gonorrhoeae*, while strategically prioritizing vaccine candidates. By exploring physicochemical traits such as molecular weight, isoelectric point, and hydrophobicity, alongside sub-cellular localization prediction, we identified potential functions of these hypothetical proteins within host cells. Utilizing specific algorithms, including those for virulence factors, our results significantly contribute to understanding the potential impact of these proteins on pathogenicity and aid in their selection as vaccine targets. Furthermore, epitope prediction methods unveil both B-cell and T-cell epitopes, offering crucial insights into the immunogenic potential of identified proteins and their capacity to stimulate protective immunity. While further experimental validation is necessary, our study establishes a foundational framework to address the pressing need for a *N. gonorrhoeae* vaccine, significantly advancing the frontiers of immuno-informatics and functional genomics. This study marks a significant step forward in leveraging computational methodologies for vaccine development, showcasing the potential of bioinformatics in addressing complex public health challenges. The nuanced insights derived from our comprehensive approach not only hold promise in the specific context of gonorrhea but also pave the way for innovative strategies in the broader landscape of infectious diseases.

## Data Availability

The original contributions presented in the study are included in the article/Supplementary Material, further inquiries can be directed to the corresponding author.
