# Supplementary material for: Artificial intelligence-driven reverse vaccinology for Neisseria gonorrhoeae vaccine: Prioritizing epitope-based candidates
Source: Front Mol Biosci. 2024 Aug 13;11:1442158. doi: 10.3389/fmolb.2024.1442158 (PMC11347834; doi:10.3389/fmolb.2024.1442158)
Supplement: Supplementary file 1 [file Table1.pdf]

Supplementary Data

Supplementary Table S1. List of bioinformatics tools used in the study

**Computational tools**	**URL**	**Year of developmen (1st release)**	**Remarks**
**Physicochemical characterization**			
ExpaxyProtParam	http://web.expasy.org/protparam/	2005	Used to calculate a variety of chemical and physical properties
**Conserved Domain and Classification**			
CDD-BLAST	http://www.ncbi.nlm.nih.gov/Structure/cdd/wrpsb.cgi	2002	The search tool for conserved domains
ScanProsite	http://prosite.expasy.org/scanprosite/	2006	Examines the motif, domain, and pattern of a protein
SMART	http://smart.embl-heidelberg.de/	1998	Identify domains in the protein
Pfam	http://pfam.xfam.org/search	1995	Utilises MSA for protein family search
InterProScan	http://www.ebi.ac.uk/InterProScan/	1999	For motif discovery
**Sub-cellular Localization**			
CELLO	http://cello.life.nctu.edu.tw	2004	Sub-cellular localization prediction. Accuracy is 91%.
CELLO2GO	http://cello.life.nctu.edu.tw/cello2go/	2014	Sub-cellular localization prediction

			Along with Gene ontology
SignalP	http://www.cbs.dtu.dk/services/SignalP/	1997	Forecasts the location of the signal peptide's cleavage.
pSORTb	http://www.psort.org/psortb	2003	Sub-cellular localization prediction
PSLpred	https://webs.iiitd.edu.in/raghava/pslpred/	2005	SVM based Sub-cellular Localization prediction
SecretomeP	http://www.cbs.dtu.dk/services/SecretomeP/	2004	predict non-classical secretion from bacteria
DeepTMHMM	https://dtu.biolib.com/DeepTMHMM	2022	Deep learning based trans-membrane helix prediction
HMMTOP	http://www.enzim.hu/hmmtop/	2001	Used for predicting transmembrane topology
**Virulence Factor**			
VirulentPred	http://bioinfo.icgeb.res.in/virulent/	2008	SVM based prediction of virulent proteins
VICMPred	http://www.imtech.res.in/raghava/vicmpred/	2006	SVM based prediction of virulent proteins from gram - bacteria
**Antigenicity Prediction**			
Vaxijen	http://www.ddg-pharmfac.net/vaxijen/VaxiJen/VaxiJen.html	2007	Antigenicity prediction
**Allergenicity Prediction**			
AllerTOP	https://www.ddg-pharmfac.net/AllerTOP/	2013	Allergenicity prediction
**Toxicity Prediction**			

ToxinPred	https://webs.iiitd.edu.in/raghava/toxinpred/index.html	2013	Toxicity prediction
**Epitope prediction, population coverage**			
NetCTLpan	https://services.healthtech.dtu.dk/services/NetCTLpan-1.1/	2010	CTL epitope prediction for 12 HLA supertypes
IEDB T-cell	http://tools.iedb.org/main/tcell/	2003	T-cell epitope prediction
Population coverage	http://tools.iedb.org/population/	2006	Population coverage prediction
Antibodyepitope	http://tools.iedb.org/bcell/	2006	Linear B-cell epitope prediction
Ellipro	http://tools.iedb.org/ellipro/	2008	Conformational B-cellepitope prediction

Supplementary Table S2. Shortlisted GDC proteins with their accession ID and UniProt ID

SL No.	Protein Accession ID	UniProt ID
1	WP_003690500.1	Q5FAK6
2	WP_010359741.1	A0A0H4ISI6
3	WP_003699500.1	A0A4D7WJT0
4	WP_003687455.1	Q9JZX6
5	WP_003690747.1	Q5F9W6
6	WP_003687711.1	Q5F9R8
7	WP_003690844.1	Q5F9K2
8	WP_010357457.1	Q5F9F7
9	WP_003689132.1	Q5F9B4
10	WP_003706020.1	Q5F9A7
11	WP_010951062.1	Q5F9A2
12	WP_010358353.1	Q5F7Z3

13	WP_225577510.1	Q5F7S3
14	WP_010951199.1	Q5F7K6
15	WP_003705520.1	Q5F7L5
16	WP_010951229.1	Q5F6B6
17	WP_225577457.1	Q9JYB9
18	WP_232469644.1	Q5F6V0
19	WP_003701667.1	Q5F6A8
20	WP_010951360.1	A0A0H4J5Y2

*GDC- Great degree of confidence

Supplementary Table S3. Probable CTL epitopes with their restricted HLA and corresponding IC_{50} values.

Source Sequence	Epitope	HLA	IC_{50} (nM)
WP_010951062 (protein_1)	VTKVKTGVY	HLA-A*30:02	40.78
		HLA-B*15:01	220.29
	RPANPDAVF	HLA-B*07:02	16.94
		HLA-C*03:03	24.75
		HLA-B*35:01	68.33
		HLA-B*15:02	93.59
	SPADVLMSL	HLA-B*07:02	20.27
		HLA-B*15:02	50.73
		HLA-C*03:03	75.96
		HLA-B*39:01	146.02

	AEDNGEDPL	HLA-B*40:01	19.18
		HLA-B*15:02	64.01
WP_010951360 (protein_2)	LSEQKIEFY	HLA-C*03:03	66.77
	YALLKTWVY	HLA-C*03:03	14.41
		HLA-B*35:01	22.26
		HLA-A*29:02	151.97
	SVVRGYFGY	HLA-A*29:02	43.73
		HLA-C*03:03	60.34
		HLA-B*15:02	68.59
		HLA-B*35:01	186.89
		HLA-A*30:02	195.19
		HLA-A*26:01	238.02
	LVIAVIASM	HLA-C*03:03	17.05
		HLA-B*15:01	49.09
		HLA-B*35:01	86.02
		HLA-A*26:01	91.75
		HLA-A*02:06	104.12
	ESVKAMLFL	HLA-C*03:03	41.36
		HLA-B*15:02	55.88
		HLA-A*68:02	125.2
	KQYAGKLGK	HLA-A*03:01	23.11
		HLA-A*30:01	58.74
		HLA-A*11:01	63.28

		HLA-C*03:03	218.08
		HLA-A*31:01	235.76
	RESGSSLGL	HLA-B*40:01	13.64
		HLA-C*03:03	19.53
		HLA-B*15:02	8.35
	MEQYGKIKL	HLA-B*15:02	23.4
		HLA-B*40:01	2.94
		HLA-C*03:03	67.24
		HLA-B*18:01	238.25
	EEIPFDLYL	HLA-C*03:03	36.11
		HLA-B*40:01	53.2
		HLA-B*15:02	99.14
		HLA-B*44:03	166.91
	KTWVYEQPY	HLA-A*32:01	43.65
		HLA-A*30:02	66.91
		HLA-A*29:02	143.47
		HLA-B*58:01	154.63
	GSIEGMEQY	HLA-C*03:03	90.70
		HLA-B*15:02	210.97
		HLA-A*29:02	225.30

Supplementary Table S4. Top fifteen epitopes from both proteins along with their corresponding immunogenicity score.

S. No.	Epitope	Immunogenicity score	Immunogenicity inference

1	VTKVKTGVY	-0.1492	Non-immunogenic
2	RPANPDAVF	0.07341	Immunogenic
3	SPADVLMSL	-0.18008	Non-immunogenic
4	AEDNGEDPL	0.14018	Immunogenic
5	LSEQKIEFY	-0.01588	Non-immunogenic
6	YALLKTWVY	0.02284	Immunogenic
7	SVVRGYFGY	0.2136	Immunogenic
8	LVIAVIASM	0.18441	Immunogenic
9	GSIEGMEQY	0.02847	Immunogenic
10	ESVKAMLFL	-0.27176	Non-immunogenic
11	KQYAGKLGK	-0.12139	Non-immunogenic
12	RESGSSLGL	-0.32599	Non-immunogenic
13	MEQYGKIKL	-0.225	Non-immunogenic
14	EEIPFDLYL	0.1554	Immunogenic
15	KTWVYEQPY	0.09985	Immunogenic

Supplementary Table S5. Antigenicity score of selected eight immunogenic peptides

S. No.	Immunogenic Epitopes	Antigenicity score
**1**	RPANPDAVF	0.3042 (Non-antigen)
**2**	AEDNGEDPL	0.1462 (Non-antigen)
**3**	YALLKTWVY	0.0086 (Non-antigen)
**4**	SVVRGYFGY	-0.1069 (Non-antigen)
**5**	LVIAVIASM	0.3216 (Non-antigen)

**6**	GSIEGMEQY	0.4244 (Antigen)
**7**	EEIPFDLYL	1.6595 (Antigen)
**8**	KTWVYEQPY	-0.0429 (Non-antigen)

Supplementary Table S6.MHC-II restricted strong binders (top one percentile) predicted from protein_1 sequence.

**HLA Allele**	**Start**	**End**	**Length**	**Core Sequence**	**Peptide sequence**	**Score**	**Percentile rank**
HLA-DRB1*07:01	19	33	15	WGYASSEAV	VKVWGYASSEAVDSD	0.9309	0.10
HLA-DRB1*07:01	18	32	15	WGYASSEAV	TVKVWGYASSEAVDS	0.8878	0.19
HLA-DRB1*15:01	18	32	15	VWGYASSEA	TVKVWGYASSEAVDS	0.8739	0.21
HLA-DRB3*02:02	360	374	15	IVKNDGTLD	FQPIVKNDGTLDDVA	0.7346	0.29
HLA-DRB1*07:01	17	31	15	WGYASSEAV	GTVKVWGYASSEAVD	0.8079	0.45
HLA-DRB1*07:01	20	34	15	WGYASSEAV	KVWGYASSEAVDSDG	0.7974	0.48
HLA-DRB1*15:01	17	31	15	VWGYASSEA	GTVKVWGYASSEAVD	0.8163	0.51
HLA-DRB3*02:02	359	373	15	IVKNDGTLD	GFQPIVKNDGTLDDV	0.6466	0.54
HLA-DRB3*02:02	361	375	15	IVKNDGTLD	QPIVKNDGTLDDVAT	0.5966	0.62
HLA-DRB1*15:01	205	219	15	IAALKAVLA	ESEIAALKAVLAKAD	0.7747	0.63
HLA-DRB5*01:01	354	368	15	LKGFQPIVK	EDPLKGFQPIVKNDG	0.5754	0.67
HLA-DRB1*03:01	69	83	15	VEDDGRTFF	EINVEDDGRTFFGAH	0.8259	0.68

HLA-DRB4*01:01	65	79	15	IEINVEDDG	GTAIEINVEDDGRTF	0.4994	0.71
HLA-DRB1*15:01	92	106	15	TGVYKGFSI	KVKTGVYKGFSIGGS	0.7451	0.72
HLA-DRB4*01:01	64	78	15	IEINVEDDG	AGTAIEINVEDDGRT	0.4744	0.84
HLA-DRB3*02:02	358	372	15	IVKNDGTLD	KGFQPIVKNDGTLDD	0.4885	0.94

Supplementary Table S7. CD4^{+} immunogenicity score for ESEIAALKAVLAKAD peptide.

Combined score	Immunogenicity score	Peptide core	Median percentile rank	HLA-DRB1 :03:01	HLA-DRB1 :07:01	HLA-DRB1 :15:01	HLA-DRB3 :01:01	HLA-DRB3 :02:02	HLA-DRB4 :01:01	HLA-DRB5 :05:01
46.82844	91.5711	IAALKAVLA	17.0	46.0	27.0	4.0	73.0	17.0	17.0	12.0

Supplementary Table S8. MHC-II restricted strong binders (top 1 percentile) predicted from protein_2 sequence.

HLA Allele	Start	End	Length	Core Sequence	Peptide sequence	Score	Percentile rank
HLA-DRB5*01:01	247	261	15	FRYTIDFDR	NGVFRYTIDFDRRKG	0.7876	0.15
HLA-DRB1*07:01	126	140	15	YEGSKGGEL	NGKKYEGSKGGELDV	0.8965	0.17
HLA-DRB1*07:01	32	46	15	VQPSVSEQL	SMSVQPSVSEQLKDN	0.8894	0.19
HLA-DRB1*07:01	127	141	15	YEGSKGGEL	GKKYEGSKGGELDVL	0.8817	0.21
HLA-DRB4*01:01	235	249	15	LSKESLDNH	NGVLSKESLDNHNGV	0.7092	0.28

HLA-DRB1*07:01	125	139	15	YEGSKGGEL	VNGKKYEGSKGGELD	0.8587	0.28
HLA-DRB1*07:01	31	45	15	VQPSVSEQL	SSMSVQPSVSEQLKD	0.8626	0.28
HLA-DRB5*01:01	246	260	15	FRYTIDFDR	HNGVFRYTIDFDRRK	0.7066	0.29
HLA-DRB4*01:01	234	248	15	LSKESLDNH	GNGVLSKESLDNHNG	0.6551	0.35
HLA-DRB4*01:01	206	220	15	IRGVATDED	LGDIRGVATDEDKLP	0.6151	0.43
HLA-DRB3*01:01	149	163	15	YGADKEQNY	KIEFYGADKEQNYAL	0.6916	0.43
HLA-DRB3*01:01	148	162	15	YGADKEQNY	QKIEFYGADKEQNYA	0.6789	0.45
HLA-DRB3*01:01	102	116	15	LFLDGEEPF	KAMLFLDGEEPFSKE	0.6699	0.49
HLA-DRB1*15:01	147	161	15	IEFYGADKE	EQKIEFYGADKEQNY	0.8187	0.50
HLA-DRB3*02:02	40	54	15	LKDNANVDA	SEQLKDNANVDAKDE	0.6299	0.55
HLA-DRB3*02:02	299	313	15	FGVNEGVAM	VSYFGVNEGVAMLEK	0.6228	0.56
HLA-DRB1*15:01	146	160	15	IEFYGADKE	SEQKIEFYGADKEQN	0.7851	0.60
HLA-DRB3*01:01	150	164	15	YGADKEQNY	IEFYGADKEQNYALL	0.6224	0.62
HLA-DRB4*01:01	205	219	15	IRGVATDED	YLGDIRGVATDEDKL	0.5181	0.64
HLA-DRB5*01:01	248	262	15	FRYTIDFDR	GVFRYTIDFDRRKGS	0.5744	0.67
HLA-DRB4*01:01	207	221	15	IRGVATDED	GDIRGVATDEDKLPK	0.5008	0.71
HLA-DRB3*01:01	101	115	15	LFLDGEEPF	VKAMLFLDGEEPFSK	0.5819	0.73
HLA-DRB3*02:02	39	53	15	LKDNANVDA	VSEQLKDNANVDAKD	0.5280	0.82

HLA-DRB1*07:01	30	44	15	VQPSVSEQL	DSSMSVQPSVSEQLK	0.7179	0.90
HLA-DRB1*15:01	173	187	15	VRGYFGYSR	YSVVRGYFGYSRKDG	0.7037	0.92
HLA-DRB1*03:01	250	264	15	IDFDRRKGS	FRYTIDFDRRKGSGS	0.7771	0.93
HLA-DRB1*03:01	251	265	15	IDFDRRKGS	RYTIDFDRRKGSGSI	0.7854	0.93
HLA-DRB3*01:01	187	201	15	IEGDGQNPE	GNPIEGDGQNPEEIP	0.4639	0.99

Supplementary Table S9.Vaxijen score, toxic and allergenic properties for Linear B-cell epitopes predicted from protein_1 and protein_2

**Protein_1**			
S. No.	**Peptide sequence**	**Vaxijen Score**	**Toxicity and Allergenic properties**
1	IAKTEAQDD	0.4819 (Antigenic)	**toxic and allergenic**
2	SSEAVDSDG	1.7093 (Antigenic)	**non-toxic and non-allergenic**
3	AIPDYMKFGAVREMHGS NAAG	0.7298 (Antigenic).	**non-toxic and non-allergenic**
4	YKGFSIGGSVTARNDLN KSQITGLKL	0.8257 (Antigenic).	**non-toxic and non-allergenic**
5	SLVDRPANPD	-0.1115 (Non- Antigenic)	**toxic and allergenic**
6	ADKPKDEAGAADKDGK PSDKPTEEEDENPK DGDKGPKTEDKGDKDA	1.6651 (Antigenic).	**toxic and allergenic**

	GKKDEAGKSASVNLSES		
7	ADKPKGGPAAKSMYQV KS	0.8436 (Antigenic).	**non-toxic and non-allergenic**
8	EDASYDNIDE	0.1334 (Non-Antigenic).	**toxic and allergenic**
9	ASEADKPADGLAAKAGK SGDLAKAESADELAKAQ DALKKSNDALAK	1.0231 (Antigenic).	**non-toxic and non-allergenic**
10	IESLKKQAVPPKGSTKAIS KAEDNGEDPLKGFQPIVK NDGTLD	0.3178 (Non-Antigenic).	**toxic and allergenic**
**Protein_2**			
S. No.	**Peptide sequence**	**Vaxijen Score**	
1	GGGSDSSMSVQPSVSEQL KDNANVDAKDEKV IEYLKKSSLKDVPKELQA KVLKVKGDEYTGVRKQ YAGKLGKGES	1.0487(Antigenic).	**toxic and allergenic**
2	EPFSKEQLQKMDVYVNG KKYEGSKGGELDVLPK GLSEQKI	0.8254(Antigenic).	**non-toxic and non-allergenic**
3	GADKEQNYALLKTWVY	0.4897(Antigenic)	**toxic and allergenic**

4	GYSRKDGNPIEGDGQNP EEIPFDLYLGDIRGVATD EDKLPKA	0.9538(Antigenic).	**non-toxic and non-allergenic**
5	GGNGVLSKESLDNHNG	0.9169(Antigenic).	**toxic and allergenic**
6	RK	NA	**toxic and allergenic**
7	IEGMEQYGKI	-0.1143 (Non-antigenic).	**toxic and allergenic**
8	AIERIPYRESGSSLGL	0.7385 (Antigenic)	**toxic and allergenic**
9	VNEGVAMLEKDNEIK	0.0961 (non-antigenic)	**toxic and allergenic**
10	EHKHQ	NA	**toxic and allergenic**
