# Supplementary material for: Artificial intelligence-driven reverse vaccinology for Neisseria gonorrhoeae vaccine: Prioritizing epitope-based candidates
Source: Front Mol Biosci. 2024 Aug 13;11:1442158. doi: 10.3389/fmolb.2024.1442158 (PMC11347834; doi:10.3389/fmolb.2024.1442158)
Supplement: Supplementary file 2 [file Image1.pdf]

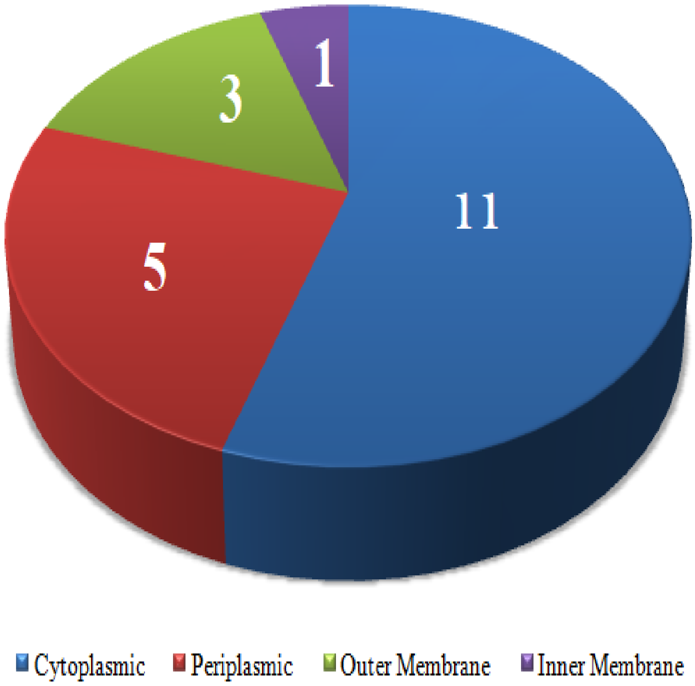

Supplementary Figure 1: Sub-cellular localization of twenty shortlisted HPs Based on the various bioinformatics tools, we predict eleven proteins to be cytosolic in nature five proteins reside in periplasm, three proteins in the outer membrane, and one protein in the inner membrane.
